# Methodological Considerations in Screening for Cumulative Environmental Health Impacts: Lessons Learned from a Pilot Study in California 

**DOI:** 10.3390/ijerph9093069

**Published:** 2012-08-24

**Authors:** Laura Meehan August, John B. Faust, Lara Cushing, Lauren Zeise, George V. Alexeeff

**Affiliations:** 1 Office of Environmental Health Hazard Assessment, California Environmental Protection Agency, 1515 Clay Street, 16th Floor, Oakland, CA 94612, USA; Email: john.faust@oehha.ca.gov (J.B.F.); lauren.zeise@oehha.ca.gov (L.Z.); george.alexeeff@oehha.ca.gov (G.V.A.); 2 Energy and Resources Group, 310 Barrows Hall, University of California, Berkeley, CA 94720, USA; Email: lara.cushing@oehha.ca.gov

**Keywords:** cumulative impacts, cumulative risk assessment, environmental justice, community health

## Abstract

Polluting facilities and hazardous sites are often concentrated in low-income communities of color already facing additional stressors to their health. The influence of socioeconomic status is not considered in traditional models of risk assessment. We describe a pilot study of a screening method that considers both pollution burden and population characteristics in assessing the potential for cumulative impacts. The goal is to identify communities that warrant further attention and to thereby provide actionable guidance to decision- and policy-makers in achieving environmental justice. The method uses indicators related to five components to develop a relative cumulative impact score for use in comparing communities: exposures, public health effects, environmental effects, sensitive populations and socioeconomic factors. Here, we describe several methodological considerations in combining disparate data sources and report on the results of sensitivity analyses meant to guide future improvements in cumulative impact assessments. We discuss criteria for the selection of appropriate indicators, correlations between them, and consider data quality and the influence of choices regarding model structure. We conclude that the results of this model are largely robust to changes in model structure.

## 1. Introduction

Environmental justice advocates and scholars have documented the disproportionate pollution burden experienced by many low-income communities of color in California [1,2,3] and elsewhere in the U.S. [4,5,6]. Polluting facilities are often concentrated in low-income communities that already face additional challenges to their health, such as limited access to health care, substandard housing, a lack of open space or recreational facilities, poor access to healthful food, and higher levels of stress stemming from poverty, under-employment or high rates of crime. This concept of “double jeopardy” [7]—the combination and potential interaction of socioeconomic stressors and elevated exposure to hazards—is absent in traditional risk assessment methods. In contrast, the evidence is growing that socioeconomic factors can increase sensitivity to the health impacts of pollution [8,9,10,11,12]. 

In a previous paper, we presented a screening method for cumulative impact assessment designed as a first step in addressing environmental justice [13,14]. Several researchers have also proposed methods to evaluate cumulative impacts [15,16,17,18]. Unlike traditional risk assessment methodologies, this method utilizes a framework that considers the presence of sensitive populations and socioeconomic factors in addition to pollution burden in accordance with the National Research Council’s call for risk assessments that consider “nonchemical stressors, vulnerability, and background risk factors” [9] and the working definition of cumulative impacts adopted by the California Environmental Protection Agency (Cal/EPA) Interagency Working Group on Environmental Justice (IWG): “Cumulative impacts means the exposures, public health or environmental effects from the combined emissions and discharges in a geographic area, including environmental pollution from all sources, whether single or multi-media, routinely, accidentally, or otherwise released. Impacts will take into account sensitive populations and socio-economic factors, where applicable and to the extent data are available.” [14].

The product of this screening method is a relative ranking of communities in California at the ZIP Code^TM^ level in regards to their potential for cumulative impacts. This method is not advanced to the stage of quantifying the probability of harm or risk. Instead, it identifies communities that warrant special attention and helps policy- and decision-makers prioritize their activities to the benefit of the most impacted communities. 

In its 2009 report, *Science and Decisions: Advancing Risk Assessment*, the National Research Council emphasized the need for “simplified risk assessment tools… (that) allow communities and stakeholders to conduct assessments and thus increase stakeholder participation” [9]. With this in mind, simplicity and transparency were key considerations in the design of this screening method. For example, only data sources available to the general public in California were used. A Cumulative Impacts and Precautionary Approaches Work Group of stakeholders from academia, industry, and civil society organizations also guided the method’s development. Some of the advice that emerged from the work group process was to quickly identify highly impacted communities in order to begin to address the problems, and to implement a scientifically-based approach that encourages meaningful public participation and incorporates contributions from the public. 

This paper considers methodological issues in combining disparate data sources related to cumulative impacts. The insights from this analysis are meant to help guide future efforts related to cumulative impacts assessment. We describe the choice of indicators and data sources, correlations between indicators and the potential for double counting. We also report on several sensitivity analyses that examine the robustness of the results to changes in the model structure and scoring regime. 

## 2. Methods

### 2.1. The Screening Method

**Figure 1:**
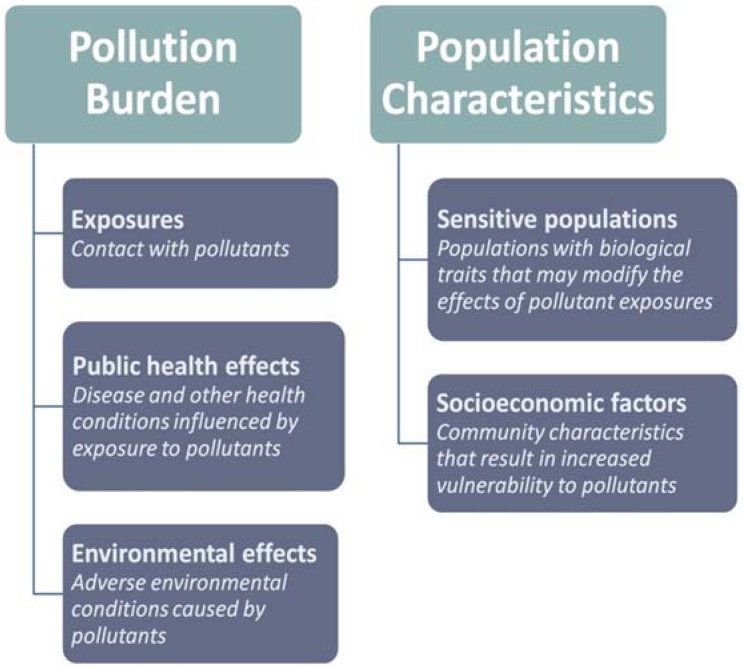
Components of cumulative impact and their definitions.

The cumulative impacts method employs a model that combines five components related to either the pollution burden or population characteristics of a geographic area. In accordance with the working definition of cumulative impacts above, the components of pollution burden are exposures, public health effects and environmental effects, and the components of population characteristics are sensitive populations and socioeconomic factors (see Figure 1). The separation of pollution burden and population characteristics allows one to distinguish between drivers of cumulative impact [15,16] and is consistent with risk assessment practices in which sensitivity factors are incorporated separately [13].

Multiple indicators were used to incorporate information about the condition of geographic areas and populations living in them for each of the components. Indicators are simple, quantitative measures of an underlying complex phenomenon. In addition to representing the phenomenon, they can also be used to guide decision-making and track progress towards a societal goal [19]. The set of indicators for a component, taken together, can be used to measure a component, and provide a relative comparison of that component for different geographies. 

**Figure 2:**
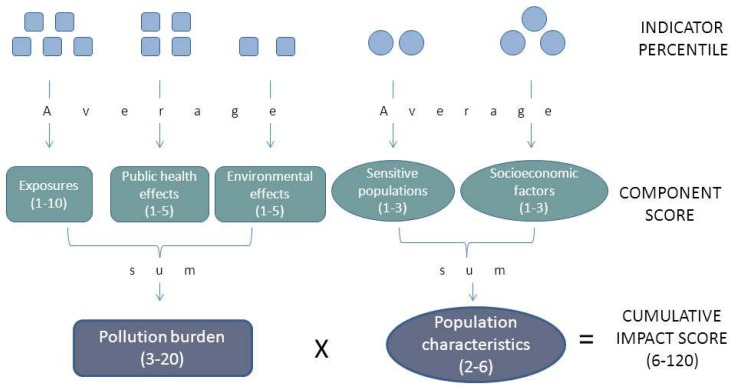
Structure of the cumulative impacts screening model.

The formula for arriving at a relative cumulative impact score from the indicators is as follows. Geographic areas are scored by their relative value of each indicator. Indicator scores are then averaged in order to arrive at a score for each component. The component scores are weighted differently to reflect certainty regarding the contribution of each component to cumulative environmental health impacts and the ability of Cal/EPA programs to address the causes of each component. The three scores for the components making up the pollution burden are summed, as are the two components making up the population characteristics. The overall cumulative impact score is the product of the scores for the pollution burden and population characteristics for each geographic area (see Figure 2).

### 2.2. Pilot Study

Thirty California ZIP Codes^TM^ were chosen to represent a diversity of geographic regions and community types. We selected indicators for this pilot analysis based on several criteria. Foremost was an indicator’s ability to represent a component of cumulative impact. Other criteria included adequate geographic resolution, enough variation in the indicator across the state to discern differences between communities, state-wide availability, currency of data, overall data quality, and our assessment of the ability of the indicator to be understood by the lay public. For the sake of transparency, we restricted ourselves to the use of publicly-available datasets. We also sought to minimize the number of indicators and the potential overlap among them in order to simplify the subsequent analysis and avoid double counting. 

Whenever possible, indicator measures for pilot ZIP Codes^TM^ were compared to those of all other ZIP Codes^TM^ in the state to derive relative indicator scores. For some data sources, the state-wide distribution was not readily available and the distribution of indicators for the 30 pilot ZIP Codes^TM^ was used instead. Indicators of exposure were assigned a score from 1–10 based on deciles; indicators of public health and environmental effects were scored from 1–5 based on quintiles; and indicators of sensitive populations and socioeconomic factors were scored from 1–3 based on tertiles. The justification of the selected range of scores for the components was described in our previous work [13]. 

### 2.3. Indicators and Data Sources

#### 2.3.1. Exposure

We chose five exposures that are important drivers of environmental health effects in California: (1) fine particulate air pollution; (2) ground-level ozone; (3) hazardous chemical emissions; (4) vehicular traffic and (5) pesticides. Ambient fine particulate matter (PM_2.5_) and ozone (O_3_) were chosen as indicators of exposures (1) and (2), respectively, and obtained from the California Air Resources Board ambient air quality monitoring network. Ambient particulate matter concentrations are reported for 24-hour intervals while ozone concentrations are reported for one- and eight-hour intervals. The air monitor closest to the population-weighted centroid was used to approximate air quality for each ZIP Codes^TM^. For PM_2.5_, the average quarterly (3-month) means were calculated for 2007 (2006 or 2005 if data was missing). For ozone, we determined the 5-year (2003–2007) average 8-hour concentration. 

Hazard-weighted pounds reported to the Toxic Release Inventory (TRI) from 2005–2007 were used as an indicator of hazardous chemical emissions in each ZIP Code^TM^. The TRI database consists of self-reported releases of over 600 toxic chemicals to air, water, land, as well as underground injection and off site transfers. The U.S. EPA’s Risk-Screening Environmental Indicators model weights TRI emissions by chemical-specific estimates of toxicity based upon the single, most sensitive chronic-health endpoint for inhalation or oral exposure, resulting in units of hazard-pounds. EPA assumes a toxicity weight of zero for chemicals with no toxicity weight available. 

For the vehicular traffic exposure indicator, the California Environmental Health Tracking Program (CEHTP) contributed data regarding on-road, mobile sources of air pollution. The data are based on year 2004 traffic counts from CalTrans’ Highway Performance Monitoring System. The sum of all unadjusted traffic volumes (vehicles per day) within a circular buffer of 2,500 meter radius around the population-weighted centroid of each ZIP Code^TM^ was obtained from CEHTP’s traffic spatial linkage web service (http://www.ehib.org/traffic_tool.jsp). 

Pesticide use data were obtained from the California Department of Pesticide Regulation’s Pesticide Use Reporting database. Pesticide use is self-reported monthly for production agricultural applications at a one square kilometer resolution and at the county level for other uses such as structural and roadsides applications. In the pilot, only production agricultural use data were used. We standardized year 2007 pounds of active ingredient applied by ZIP Code^TM^ area. 

#### 2.3.2. Public Health Effects

We developed indicators for four health outcomes with strong evidence of an environmental etiology: low birth weight, heart disease and cancer (at the ZIP Code^TM^ level) and asthma (at the county level). For the first three indicators, three-year averages for 2006–2008 were used to address inter-annual instability in estimates due to low counts in some of the geographic areas. Natality data is acquired by the California Department of Public Health (DPH) via birth certificates. We defined low birth weight as <2,500 grams and divided the number of low birth weight babies by the total number of live births in each ZIP Code^TM^ to get an annual rate. 

We included cause-specific mortality measures as indicators of heart disease and cancer based on DPH data on the primary cause of death acquired via death certificates. Age-adjusted rates were not publicly available, so we derived crude estimates using the U.S. Census year 2000 total population for each ZIP Code^TM^. 

Age-adjusted asthma hospitalization rates for 2009 were obtained for all California counties from CEHTP. Asthma hospitalizations are selected based on primary discharge diagnosis coding of inpatient discharge records provided by hospitals to the Office of Statewide Health Planning and Development. CEHTP uses county population data from the California Department of Finance to calculate a rate from counts and performs age-adjustment using the U.S. Census 2000 population. Because the data were only available at the county level, ZIP Codes^TM^ were assigned the value for the county in which they fell and scored was assigned based upon the county distribution.

#### 2.3.3. Environmental Effects

We chose two indicators of environmental degradation: hazardous sites and hazardous spills/leaks. Hazardous waste facilities and brownfields pose an exposure threat to communities via contaminated soil, water and air and can be an indicator of community blight. The location of permitted hazardous waste facilities and clean-up sites, including state and federal Superfund and military sites, was obtained from the California Department of Toxic Substances Control’s (DTSC) online Envirostor database. Each site category was given a weight of 1, 2, or 3 based their status (cleaned-up, pending, or active), and a weighted sum of all facilities within each ZIP Code^TM^ was calculated. 

Spills and leaks of hazardous materials are another threat to water quality and the overall health of the environment. Data on sites that threaten water quality were obtained from the Geotracker database maintained by the State Water Resources Control Board. Geotracker includes the geographic location of permitted and leaking underground storage tanks, spill clean-up sites, landfills, and military sites. Each site was given a weight of 1 or 3 based on its clean-up status to obtain a weighted sum of all sites within a ZIP Code^TM^. 

#### 2.3.4. Sensitive Populations and Socioeconomic Factors

Children and the elderly were chosen as indicators of sensitive populations. All data for the age and socioeconomic indicators came from the 2000 U.S. Census. The U.S. Census creates its own geography, the ZIP Code Tabulation Areas (ZCTAs^TM^), to address the issue of changing ZIP Code^TM^ boundaries by delineating areas by their most commonly occurring ZIP Code^TM^. For simplicity, we assumed no change in ZIP Codes^TM^ boundaries for our other data sources, and chose to assume equivalence between ZCTAs^TM^ and ZIP Codes^TM^ in order to make use of Census data. Age has been shown to modify susceptibility to environmental contaminants, with children and the elderly being generally more sensitive. The proportions of the population that are under age 5 and over age 65 were chosen as two indicators of sensitive populations. 

#### 2.3.5. Socioeconomic Factors

Income and education are important in determining the resources and capacity of a community to address local threats to environmental health. The percent of residents 25 and older with less than high school education and the median household income were chosen as two indicators of overall neighborhood socioeconomic status. The percent of residents with incomes less than twice the national poverty level was chosen as an additional indicator of individual-level economic deprivation. 

### 2.4. Correlations and Sensitivity Analyses

In order to examine the potential for double-counting or redundant indicators, a correlation analysis explored the strength and direction of the relationship between indicators within each component. Spearman’s rank correlation coefficient was calculated because it does not require that the data be normally distributed. 

We also undertook a sensitivity analysis to evaluate the robustness of the model results to changes in the equation used to combine indicators and the scoring regime. The model used was compared to alternative models with modifications to the range of values assigned to each component, the structure of the equation, or both.

The results were compared by examining the frequency and magnitude of changes in the ranking order. Because we are primarily concerned with identifying the most impacted communities, we specifically evaluated changes among the highest ranked communities.

## 3. Results and Discussion

**Figure 3:**
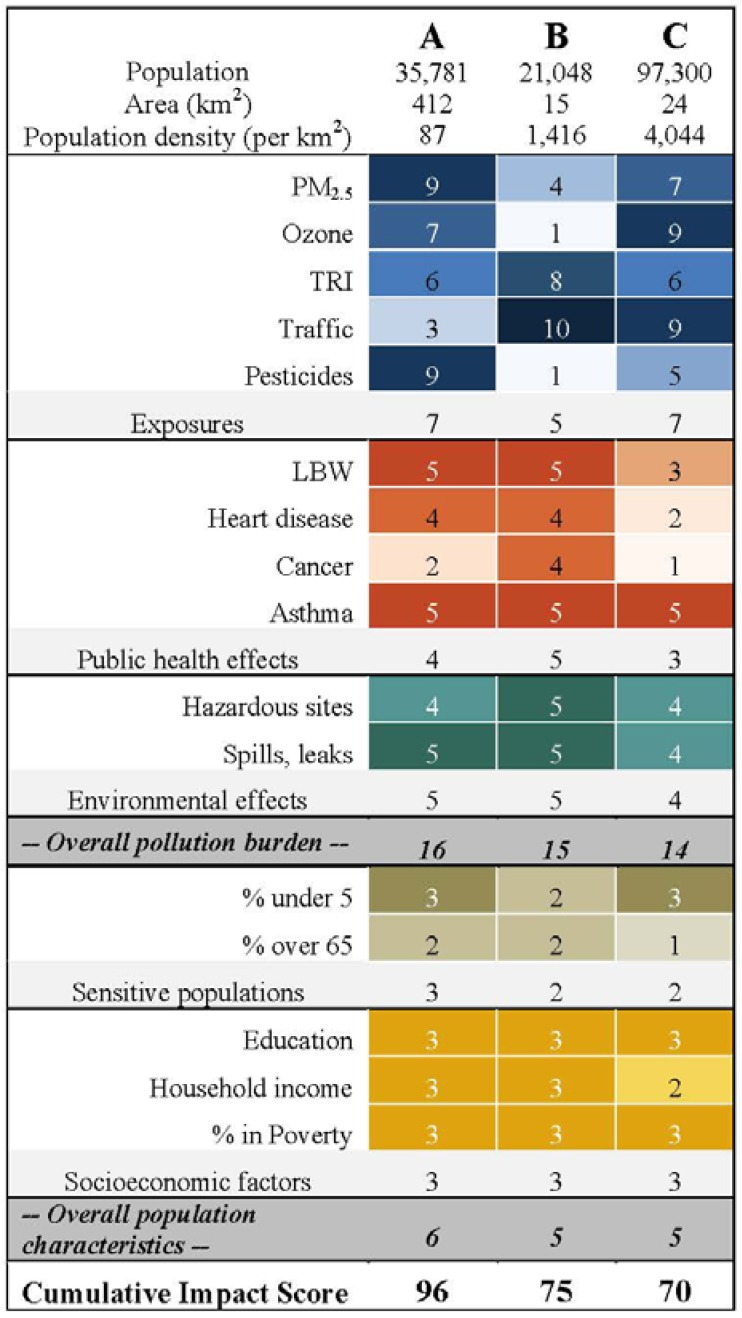
Calculation of the cumulative impact score for three sample communities.

Descriptive statistics related to the 30 pilot ZIP Codes^TM^ and the indicators are given in Table 1. Out of a possible range of 6–120, cumulative impact scores ranged from 24–96 with a median of 50 and standard deviation of 18. The component scores for the 30 ZIP Codes^TM^ are provided elsewhere [9]. Here, we provide more detail on three example communities in Figure 3. Since this screening method is primarily concerned with identifying potential environmental justice communities that warrant further investigation, we focus on high scoring communities. Because the results are currently preliminary and only illustrative of the methodology, the identity of the ZIP Codes^TM^ is kept anonymous here.

**Table 1:** Indicators included in the pilot analysis (n = 30).

	**ZIP Codes^TM ^**	**MEAN (SD ^1^) **	**MEDIAN **	**RANGE **
	Total population	30,144 (20,989)	23,472	1,793–97,300
	Area (km^2^)	341 (831)	35	2–3,678
	Population density (per km^2^)	1,795 (3,082)	474	4–15,403
	**Indicators **	**MEAN (SD ^1^) **	**MEDIAN **	**RANGE **
Pollution burden	***Exposures ***			
	PM_2.5_ (µg/m^3^)	12.1 (4.5)	10.7	5.6–20.4
	Ozone (ppb)	60.2 (12.9)	61.3	39.3–92.0
	Toxic industrial emissions (hazard-lbs)	4.9 × 10^10^ (2.7 × 10^11^)	1.4 × 10^5^	0–1.5 × 10^12^
	Traffic volume (vehicles x day^−1^ × 1,000 km^−2^)	16,457 (16,359)	8,742	909–67,285
	Pesticide use (lbs active ingredient/km^2^)	1,710 (3,718)	13	0–16,948
	***Public health effects***			
	Low birth weight rate (% of live births <2,500 g/year)	7.0 (1.5)	7.2	4.0–10.3
	Heart disease mortality rate(deaths per 100,000 per year)	194 (65)	197	79–338
	Cancer mortality rate(deaths per 100,000 per year)	159 (57)	149	53–260
	Asthma hospitalization rate (per 100,000 per year)	9.7 (2.8)	10.1	3.5–15.0
	***Environmental effects***			
	Hazardous sites (EnviroStor score)	28 (37)	12	0–173
	Spills and leaks (GeoTracker score)	103 (104)	77	6–565
Population	***Sensitive populations***			
	% under age 5	7.3 (2.7)	6.9	2.9–11.4
	% over age 65	11.1 (4.9)	10.5	3.8–21.7
	***Socioeconomic factors***			
	% over age 24 with less than a high school education	30.7 (23.3)	22.2	4.3–76.4
	Median household income ($)	45,978 (24,906)	37,073	21,124–119,147
	% residents below twice the federal poverty level	40.3 (22.5)	37.3	7.8–79.3

^1^ Standard deviation.

### 3.1. Examples of High-Scoring Communities

Community A is on the edge of a large urban area that borders agricultural land in the San Joaquin Valley of California. It had the highest cumulative impact score of our 30 pilot ZIP Codes^TM^. The population has a high proportion of Hispanic/Latino and African American residents. The exposure score for this community fell in the 70th percentile and the public health effect score fell in the 80th percentile. Pesticides and air pollution contribute most to exposure; there is relatively little traffic in the immediate vicinity.

This is congruent with the fact that meteorological conditions in the San Joaquin Valley accumulate and trap air contaminants originating elsewhere. The low birth weight and asthma hospitalization rates are both in the highest quintile. Epidemiological studies have found an association between both of these health outcomes and air pollution [20,21].

Community A also contains many hazardous and clean-up sites that may imperil environmental quality, and the environmental effects score is in the 80th percentile. Finally, the community scores are high for nearly all indicators of sensitive populations and socio-economic factors. Thus the community exhibits the “double jeopardy” of high pollution burden coupled with population characteristics that may make communities more vulnerable to the health effects of contamination. 

Community B is located in a coastal urban area and was among the five highest scoring communities in our pilot analysis. It is more densely populated than Community A and is a primarily African American community. Despite high traffic and TRI emissions, ambient air quality as captured by our two indicators is good or fair, and the overall exposure component score is average. This may be an artifact of the fact that the nearest air monitor was located closer to the coast. Agricultural pesticide use is in the lowest percentile because this is an urban area. Nevertheless, this community scores nearly as high as Community A in pollution burden because of high public health and environmental effects. Part of the rationale in incorporating both of these components was to capture potential environmental health effects that are not adequately captured in existing environmental monitoring systems of background ambient conditions. 

Community C is a densely populated inland urban community in Southern California that is primarily Hispanic/Latino and was among the eight highest scoring communities in our pilot analysis. In this community, the public health and environmental effects contribute most in driving the high pollution burden score. Traffic and air pollution contribute most to the community’s sources of exposure, with only moderate TRI emissions and agricultural pesticide use. Asthma rates are high and the low birth weight rate is moderate, while the mortality measures are low. This may be related to the fact that age-standardized rates were not used, and this community has a younger age distribution. The community is home to many hazardous sites and its environmental effect score is in the 80th percentile. Finally, this community exhibits an average median household income but large percentage of people living in poverty. This is possible if there is great disparity in household income within a community, and illustrates the potential importance of including both indicators. 

### 3.2. Indicator Correlation

Pearson’s correlation coefficients for the 16 indicators are shown in Table 3 (n = 30 ZIP Codes^TM^). The most strongly correlated indicator pairs were: the two indicators of environmental effects (ρ = 0.77); heart and cancer mortality (ρ = 0.72); the mortality measures and % over 65 (ρ = 0.73 for heart and 0.77 for cancer mortality); and all combinations of the three indicators of socioeconomic vulnerability (0.72 ≤ |ρ| ≤ 0.94). 

It is possible that the environmental effects indicators are correlated because of overlap between the two databases, but it is also reasonable to assume that industrial and clean-up sites are in fact spatially clustered. Future analyses will examine the issue of double-counting by evaluating the degree of overlap between databases. The correlation between mortality measures, as well as their correlation with age, is most likely a product of the fact that age-standardization was not applied. Age-standardization would improve the mortality measures in terms of identifying potential environmental contributions to disease burden.

The socioeconomic indicators were highly correlated (|ρ| ≥ 0.72). We believe inclusion of multiple indicators of socioeconomic status is appropriate despite their correlation because they measure subtle differences between communities. For example, neighborhoods with similar median household income may have different proportions of residents living in poverty depending on the degree of income disparity.

This analysis also reveals that traffic and pesticide use are inversely correlated (ρ = −0.63), as would be expected given the predominance of one in urban areas and the other in rural. The two age indicators are also strongly negatively correlated, indicating a “canceling out” effect. That is, communities with a high proportion of young children do not tend to have a high proportion of older individuals. This may have a homogenizing effect on the overall scores of sensitive populations (creating many scores of 2), and a single indicator that encompasses both children and elderly may be more appropriate. 

It is also interesting to note that the exposure indicators were only moderately correlated with public health effects (|ρ| < 0.54) or environmental effects (|ρ| < 0.43). To the extent that public health and environmental effects reflect pollution burden, this analysis thus suggests their inclusion adds important additional information.

### 3.3. Sensitivity Analysis

**Figure 4:**
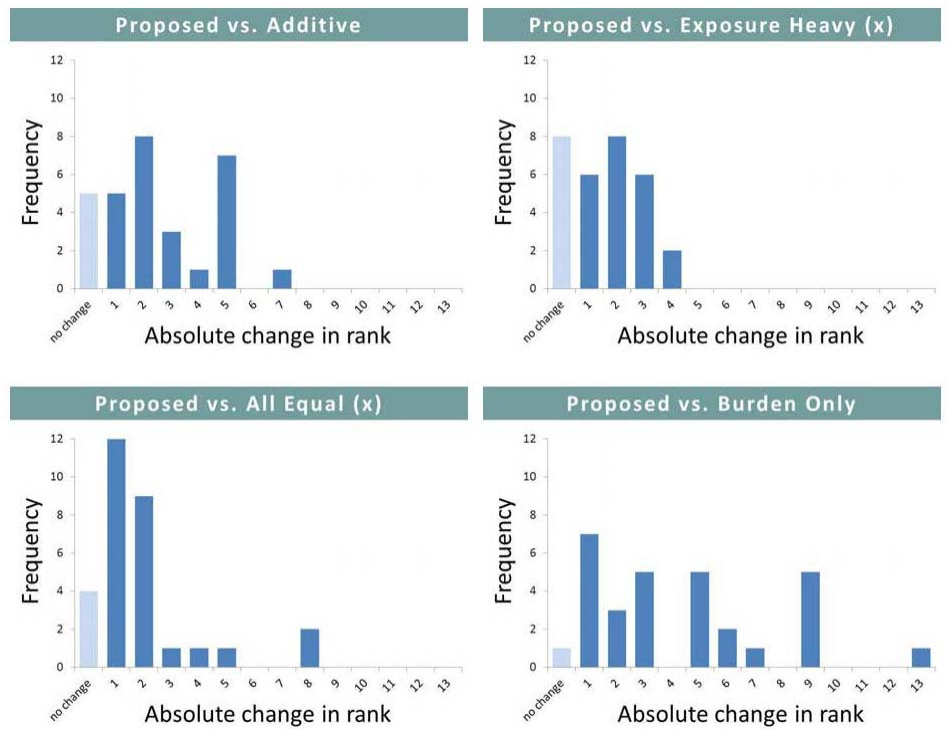
Frequency and magnitude of changes in rank associated with varying the model.

Six alternative models were evaluated against our proposed model (see Table 2). Among the communities with the highest impact (top six), changing the model resulted in few changes (see Table 4). The magnitude and frequency of overall changes in rank however differed considerably among the various models (see Figure 4). The “burden only” model resulted in the greatest number of total changes (14) as well as the greatest number of changes in rank over six or more positions (9). Our scoring regime appeared to have moderate to little effect on the overall results, with both “all equal” models resulting in only 5 changes in the ranking order of communities. None of these were among the highest ranking communities.

**Table 2:** Alternative models used in the sensitivity analysis ^1^.

Model	Equation	Component Scoring Scheme	Maximum Possible Score
Proposed	(A + B + C) × (D + E)	(10 + 5 + 5) × (3 + 3)	120
Additive	(A + B + C) + (D + E)	(10 + 5 + 5) + (3 + 3)	26
Exposure Heavy (×)	(A + B + C) × (D + E)	(20 + 5 + 5) × (3 + 3)	180
Exposure Heavy (+)	(A + B + C) + (D + E)	(20 + 5 + 5) + (3 + 3)	36
All Equal (×)	(A + B + C) × (D + E)	(10 + 10 + 10) × (10 + 10)	600
All Equal (+)	(A + B + C) + (D + E)	(10 + 10 + 10) + (10 + 10)	50
Burden Only	(A + B + C)	(10 + 5 + 5)	20

^1^ A = exposure; B = public health effects; C = environmental effects; D = sensitive populations; and E = socioeconomic factors.

**Table 3:** Pearson’s correlation coefficients between indicators (n = 30). Absolute values ≥0.6 are highlighted in bold.

	Exposure					Public Health Effects				Environmental Health Effects		Sensitive Populations		Socioeconomic Factors		
	PM_2.5_	Ozone	TRI	Traffic	Pesti-Cides	LBW	Heart	Cancer	Asthma	Hazard-Ous Sites	Leaks and spills	Under 5	Over 65	Education	Median Income	Poverty
PM_2.5_	1.00															
Ozone	0.58	1.00														
TRI	0.10	−0.11	1.00													
Traffic	0.12	−0.21	−0.02	1.00												
Pesticides	0.09	0.23	0.20	**−0.63**	1.00											
LBW	−0.13	0.06	−0.09	0.23	−0.24	1.00										
Heart	−0.04	0.01	−0.24	−0.07	−0.34	0.02	1.00									
Cancer	−0.33	−0.37	−0.15	−0.04	−0.44	−0.08	**0.72**	1.00								
Asthma	0.43	0.28	0.53	0.21	0.07	0.05	−0.17	−0.40	1.00							
Hazardous sites	−0.24	−0.09	0.42	0.15	0.05	0.18	−0.09	−0.08	0.34	1.00						
Leaks and spills	−0.12	−0.06	0.32	0.32	−0.12	0.35	−0.08	−0.17	0.39	**0.77**	1.00					
Under 5	0.31	0.26	0.44	−0.19	0.46	−0.10	−0.51	**−0.68**	0.35	0.27	0.19	1.00				
Over 65	−0.33	−0.31	−0.45	0.13	−0.50	0.17	**0.73**	**0.77**	−0.33	−0.11	−0.05	**−0.80**	1.00			
Education	0.22	0.14	0.38	−0.21	0.45	−0.16	−0.38	−0.45	0.31	0.29	0.19	**0.81**	**−0.65**	1.00		
Median income	−0.25	−0.14	−0.40	0.06	−0.22	0.07	0.25	0.31	−0.48	−0.48	−0.39	**−0.60**	0.50	**−0.72**	1.00	
Poverty	0.30	0.16	0.39	−0.13	0.36	−0.15	−0.35	−0.42	0.41	0.35	0.28	**0.73**	**−0.63**	**0.89**	**−0.94**	1.00

### 3.4. Limitations and Challenges

Several challenges arose in our effort to combine disparate data sources that are likely to be confronted by others conducting cumulative impact assessments. The choice of geographic level of analysis is always difficult because data are available in different forms. We chose ZIP Codes^TM^ because we felt they were of a size appropriate to capture a “community” and would be more easily interpreted by the public and decision-makers. We also wanted to avoid implying greater geographic precision than appropriate for the data sources that had coarse geographic resolution. However, ZIP Code^TM^ boundaries change according to the needs of the U.S. Postal Service, and geographic boundary files must be purchased from private vendors. Moreover, Census data are released as ZCTA^TM^ rather than ZIP Code^TM^ estimates. For simplicity, we therefore chose to assume perfect geographic overlap between ZCTAs^TM^ and ZIP Codes^TM^ although they in fact vary. 2010 ZCTAs^TM^ better correspond to ZIP Codes^TM^ and also exclude large uninhabited areas; using 2010 Census data will therefore serve as an improvement. 

Missing data was one challenge that is of particular concern for the public health indicators, where data need to be suppressed for confidentiality reasons and where low counts produce unstable estimates that vary substantially year-to-year. We took multi-year averages to help address this problem. We also chose to use three-year averages for the ambient air monitoring data to account for discontinuous monitoring and to minimize the contribution of extreme weather patterns.

Many important facets of environmental and public health are not monitored statewide and data for those available statewide may not be in an easily accessible data source or format. For example, most of our exposure indicators are related to air pollution and we are working to identify indicators that address other exposure pathways. Not all health data were accessible at the ZIP Code^TM^ geographic resolution nor in the form of age-adjusted rates. Cancer incidence rather than mortality would also arguably be a better measure because many people with cancer die of other causes. In the future, we plan to request additional health data and consider sources of information not contained in this pilot. However, even in the face of data gaps and limitations we feel that it is important to move forward with using, as well as refining the data that is available in order to begin to address the needs of environmental justice communities. 

Our method was developed to conform with the Cal/EPA definition of cumulative impacts. In this way indicators of public health effects such as low birth rate were considered to be reflective of pollution burden. However, we recognize that the public health effects indicators we identified could also have served as indicators of population characteristics. Public health outcomes may also be considered as a combination of pollution burden and population characteristics. For example, our indicator, asthma hospitalization rate, can reflect environmental exposures to air pollution as well as the ability to manage and cope with asthma symptoms which may be reflective of socioeconomic status. 

Another challenge we encountered is in setting the relative scores or weights for the components and indicators. However, concern over this challenge is tempered because our overall goal is to identify the most impacted communities and not to discern small differences amongst communities. As shown as Table 4, the identification of most impacted communities is insensitive to the alternative model structures and weights.

**Table 4:** Changes in rank associated with varying the model.

Model	Changes among the Six Most Impacted Communities	Total Changes (out of 30)
Proposed *vs*. Additive	0	10
Proposed *vs*. Exposure Heavy (×)	1	9
Proposed *vs*. Exposure Heavy (+)	1	12
Proposed *vs*. All Equal (×)	0	5
Proposed *vs*. All Equal (+)	0	5
Proposed *vs*. Burden Only	0	14

There is also no clear way in which to validate the results of our analysis because there is no definitive measure of “cumulative impact”. Going forward, we consider acceptance and use of the method by the regulators and environmental justice community-based organizations that will use it as an important indication of its quality and utility. With the identification of communities that score high, more detailed evaluation of communities can be performed to better understand how intervention efforts may be best targeted. Comparing areas identified as impacted by our screening method with other methods, such as the Environmental Justice Screening Method or the Cumulative Environmental Vulnerability Assessment, for example, will provide additional information on the strength of our method [15,16].

## 4. Conclusions

This pilot study successfully applied a cumulative impacts screening method to 30 communities in California. Using existing data, communities in California of high potential environmental justice concern were identified. The issues confronted when considering such a wide range of different data sources provides insight into ways to improve the way data are generated and collected. The ability to consistently identify the same communities as most impacted regardless of the model structure supports the use of this screening method for relative ranking. Only publicly available sources of data were used in this analysis in order to allow for maximum transparency. However, this resulted in several limitations in regards to specific indicators. Future work will include working with colleagues and stakeholders to improve each indicator and scaling up the analysis to the entire state. 
